# Post-status epilepticus treatment with the Fyn inhibitor, saracatinib, improves cognitive function in mice

**DOI:** 10.1186/s12868-020-00606-z

**Published:** 2021-01-15

**Authors:** Xin-Ming Luo, Jing Zhao, Wen-Yue Wu, Jie Fu, Zheng-Yu Li, Ming Zhang, Jie Lu

**Affiliations:** 1 Department of Neurology, The Second Affiliated Hospital of Nanchang University, No. 1 Minde Road, Nanchang, 330006 Jiangxi China; 2 Institute of Neuroscience, Nanchang University, Nanchang, 330006 Jiangxi China

**Keywords:** Status epilepticus, Fyn inhibitor, Saracatinib, Cognitive function, Hippocampal neurons

## Abstract

**Background:**

Status epilepticus (SE) is a life-threatening neurological disorder. The hippocampus, as an important area of the brain that regulates cognitive function, is usually damaged after SE, and cognitive deficits often result from hippocampal neurons lost after SE. Fyn, a non-receptor Src family of tyrosine kinases, is potentially associated with the onset of seizure. Saracatinib, a Fyn inhibitor, suppresses epileptogenesis and reduces epileptiform spikes. However, whether saracatinib inhibits cognitive deficits after SE is still unknown.

**Methods:**

In the present study, a pilocarpine-induced SE mouse model was used to answer this question by using the Morris water maze and normal object recognition behavioral tests.

**Results:**

We found that saracatinib inhibited the loss in cognitive function following SE. Furthermore, we found that the number of hippocampal neurons in the saracatinib treatment group was increased, when compared to the SE group.

**Conclusions:**

These results showed that saracatinib can improve cognitive functions by reducing the loss of hippocampal neurons after SE, suggesting that Fyn dysfunction is involved in cognitive deficits after SE, and that the inhibition of Fyn is a possible treatment to improve cognitive function in SE patients.

## Background

Epilepsy is a chronic neurological disorder characterized by a persistent occurrence of seizures. Status epilepticus (SE) is characterized by prolonged seizures or intermittent seizures and unconsciousness [1], which poses a serious threat to life, and results in high mortality rates [2]. A growing body of evidence suggests that SE is associated with damage to the hippocampus. It has been reported that cognitive deficits often occur in patients with SE and in experimental SE models. Cognitive deficit, as a long-term sequelae followed by SE [3], usually result from hippocampal neuron loss. Neuronal loss in the hippocampus often results from apoptosis or aberrant neurogenesis [4]. Rescuing the loss of hippocampal neurons may therefore be an effective treatment to improve cognitive function after SE.

Fyn, a member of the Src family of kinases, is a tyrosine kinase without a receptor. It is widely involved in many physiological processes in the nervous system, including synaptic transmission, synaptic plasticity, oligodendrocyte differentiation, and dendritic spine development [5–8]. It also plays critical roles in memory formation and cognitive function regulation by regulating hippocampal dendritic spine development in Alzheimer’s disease (AD) patients [7]. Aberrant Fyn activity leaded to speed up neurodegeneration in AD patients, and deficiency of Fyn or inhibiting its activity has been shown to restore memory function in AD mouse model [9, 10]. Moreover, a previous study reported that inhibition of Fyn prevented spatial memory deficits caused by intraventricular hemorrhage [11]. However, whether inhibition of Fyn involved in cognitive deficits remains unclear. Fyn in neuron is known to modulate both NMDA and GABA_A_ receptors and is associated with both excitatory and inhibitory ion channels, which are potentially associated with the onset of seizure [5, 12, 13]. Silencing of the *Fyn* gene reduced oligodendrocyte apoptosis in an epileptic model in vitro [14], and previous studies have suggested that Fyn is closely related to epilepsy. Thus, inhibiting Fyn may be a possible treatment for epilepsy. Saracatinib, a pharmacological inhibitor of Fyn, is usually used to treat AD patients [15–17]. It was shown to be effective at suppressing epileptogenesis and reducing epileptiform spikes and spontaneous convulsive seizures in a rat SE model [18]. However, whether it improves cognitive function deficits after SE is unclear. To answer this question, we characterized possible pathological changes in the hippocampus after SE, using the pilocarpine-induced SE model in mice.

## Methods

### Animals

C57BL/6 mice (all males, 3 months of age) were purchased from the Animal Center of Nanchang University and used in this study. All mice were maintained under a 12:12 h light:dark cycle and had free access to food and water. All experiments were performed in accordance with international guidelines of animal experimentation (ARRIVE guidelines) and were approved by the Animal Care Committees of The Second Affiliated Hospital of Nanchang University.

### Induction of SE

SE was induced in mice according to a previous report [19]. Briefly, mice were injected with a low dose of pilocarpine hydrochloride (100 mg/kg) (Sigma Aldrich, St. Louis, MO, USA) by intraperitoneal (i.p.) administration every 20 min until the onset of SE. Methylscopolamine nitrate (1 mg/kg; i.p., Sigma Aldrich) was injected 30 min before the injection of pilocarpine to alleviate peripheral cholinergic side effects. A similar volume of 0.9% (w/v) sterile saline was injected into control mice. SE was defined as continuous stage 4 (forelimb clonus and rearing) or 5 (forelimb clonus with rearing and falling) seizure activities according to the Racine scale [20]. The first seizure usually occurred after three injections. If SE was not induced after five injections, no further attempts were made to avoid death of the mouse. All mice that developed SE lasting for an hour and a half hours received diazepam (10 mg/kg) to terminate seizure activity. Control mice were also injected with the same dose of diazepam. Mice that did not develop SE and SE lasting less than 1.5 h, and died after pilocarpine injections were excluded. Mice were distributed into three groups: the developed SE group (SE group); Saracatinib (Sar) treatment group (Sar + SE group); and the control group (CON group).

### Behavioral testing

**Fig. 1:**
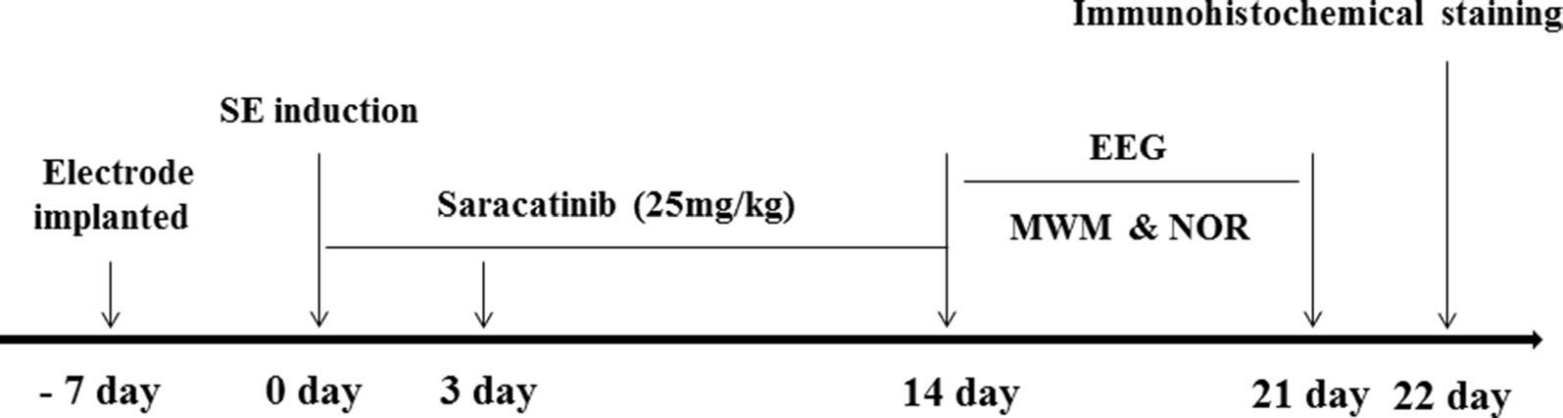
Time depiction of the experimental scheme

Mice cognitive functions was assessed using the Morris water maze (MWM) and novel object recognition (NOR) behavioral tests. The investigator was blinded to the genotype during testing. Data were analyzed using Smart v2.5.21 from Panlab. The experimental scheme is shown in Fig. 1.

### MWM

A circular pool with a diameter of 120 cm and a depth of 50 cm was used. Water was filled with the pool until the platform (10 cm diameter) is 1 cm under the water surface. The water temperature was kept at 22 °C during testing. Mice were tasked to find a target platform within 90 s from different starting positions in each trial. Four trials were performed each day at least 30 min interval time for five consecutive days. Mice were allowed to stay on the platform for 20 s. Escape latencies (time spent swimming from the start point to the target platform) and path length (the distance from start point to the target platform) before reaching the target platform were recorded on these five consecutive days. On day 6, the probe trial was performed to assess memory consolidation by removing the target platform. The number of area crossings in the target platform placed in previous training and the staying time in the quadrant of the target platform placed in previous training were recorded (n = 9 in CON group; n = 8 each in the SE group and Sar + SE groups).

### Novel object recognition test

Mice were exposed to two identical objects placed at a distance of 10 cm from the sidewalls in two opposite corners of the apparatus for 10 min in the open field (60 cm × 60 cm) in the training session. After 90 min, the mice were allowed to explore in the presence of one familiar and one novel object for 10 min. Mice that unhabituated (lasting froze more than one min or always away from objects) were excluded in exploring session. The preference index (PI) was defined as the time exploring one of the identical objects/total time exploring two of the identical objects. Recognition index (RI) was defined as the time exploring the novel object/total time exploring the novel object and the familiar object. [n = 9 each in the CON group and SE group; n = 10 in the Sar + SE groups].

### Drug administration

Sarscatinib (dissolved in saline) was administered (25 mg/kg) orally starting 2 h after diazepam injection and repeated twice daily for the first 3 days followed by a single dose each day for the next 11 days during the 2 weeks after SE. Same volume of saline was gave to CON mice.

### Electroencephalogram (EEG) recording

Mice that received continuous video-EEG monitoring were implanted with silver wire electrodes (0.125 mm in diameter) into the hippocampal dentate gyrus (DG) region after anesthesia with pentobarbital (Sigma Aldrich, 50 mg/kg, i.p.). The electrode implantation site used the following coordinates with the bregma as the reference: bregma: − 2.3 mm, lateral: 1.8 mm and depth: 2.0 mm. The reference electrode was placed in the frontal cortex. All implanted surgery was performed at 7 days before the induced SE. EEG activity was recorded 12 h every day for up to 7 days at 14 days after induced SE using PowerLab 8/35 software (ADInstruments, Sydney, Australia) (High-pass filter, 0.3 Hz cutoff; low-pass filter, 100 Hz). Epileptic spikes (sharp (< 50 ms) positive or negative deflections with amplitudes exceeding twice the baseline EEG) were detected and scored by the Gotman spike using PowerLab software. Mice behavior was monitored using video and reviewed by an investigator who was blinded to the identity of the groups (n = 5 each in every group).

### Immunohistochemistry staining

One month after induced SE, the mice were anesthetized with pentobarbital (Sigma Aldrich; 50 mg/kg, i.p.) and transcardially perfused with phosphate-buffered saline (PBS) followed by 4% paraformaldehyde in 0.1 M PBS to sacrifice the mice. The brain was dissected and fixed in 4% paraformaldehyde overnight at 4 °C and then cryoprotected in 30% sucrose in PBS for 72 h at 4 °C. Coronary sections (8 μm) were cut with a cryostat and mounted onto glass slides. Sections were washed in PBS and rehydrated in ethanol of decreasing concentrations. After washing with PBS, the sections were incubated with anti-NeuN antibody (ab104224, 1:500; AB_10711040; Abcam, Cambridge, UK) overnight at 4 °C and washed with PBS. The sections were then incubated with secondary antibody and visualized using 3,3-diaminobenzidine. NeuN-positive neurons in a 100 μm line long DG of hippocampus were counted in five sections from identical levels in each mouse (n = 5/groups).

### Statistical analysis

Data are expressed as the mean ± SEM. One-way or two-way analysis of variance was used to assess differences between two groups and the least significant difference (LSD) or Dunnett T3 post hoc test was used to compare multiple groups after normality tests. A value of *p* < 0.05 was considered statistically significant.

## Results

### Mice behavioral characteristics after pilocarpine injection

Mice often exhibited hypoactivity, curling up, tremors, head bobbing, and myoclonic movement of the limbs after pilocarpine injection. SE usually began by rearing with or without falling and jumping at the onset of SE.

### Saracatinib reduces the frequency of epileptic spikes after SE

**Fig. 2:**
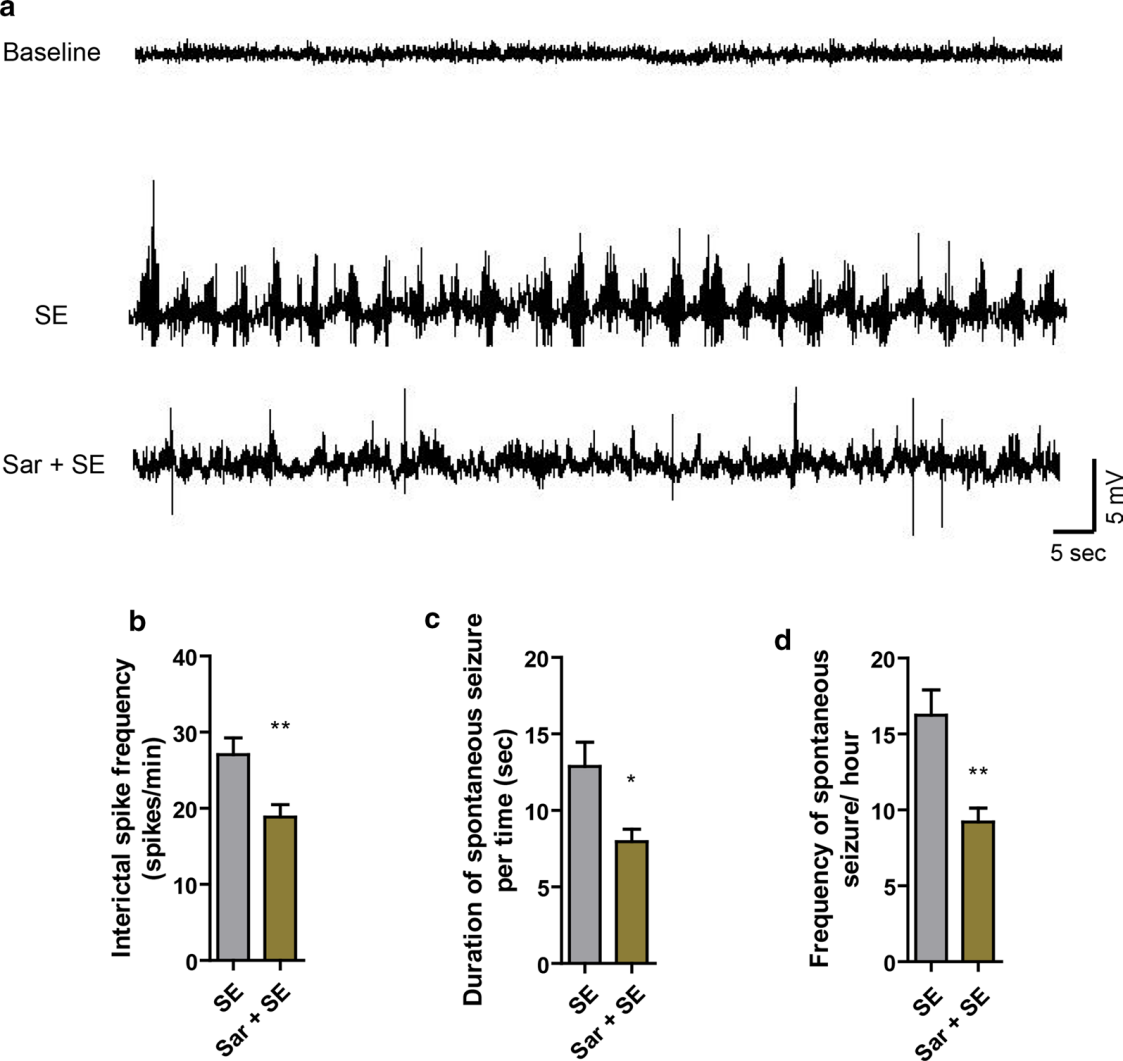
Saracatinib treatment reduces the frequency of epileptic spikes after status epilepticus (SE). **a** Representative electroencephalogram traces from the hippocampus 14 days after SE. **b** Quantitative analysis of interictal spikes. **c** Quantitative analysis of duration of spontaneous seizure. **d** Quantitative analysis of frequency of spontaneous seizure. Data are expressed as the mean ± SEM (n = 5/group). **p < 0.01

Saracatinib, as a pharmacological inhibitor of the Fyn, is effective at suppressing epileptiform spikes [18]. We therefore examined the effects of saracatinib on the frequency of spikes using our pilocarpine induced SE model. We found that post-SE saracatinib injection decreased the frequency of epileptic spikes in the hippocampus (Fig. 2b) (*t*_*(8)*_=2.986*, p *=0.007). Furthermore, video-EEG showed that saracatinib injection decreased the duration (Fig. 2c) (*t*_*(8)*_=2.763*, p *=0.025) and frequency (Fig. 2d) (*t*_*(8)*_=3.715*, p *=0.006) of spontaneous seizure after SE (Table 1). These results suggested that our protocol was successful at inducing SE, and that saracatinib injection was effective at relieving SE.

**Table 1:** Characteristics of the mice with SE

Groups	Number	Duration of SE (min)	Mice surviving SE	Mice developing spontaneous seizure after SE	Duration of spontaneous seizure per time (s)	Frequency of spontaneous seizure per hour
CON	30	0	30	0	0	0
SE	36	90	22 (61.1%)	22 (100%)	12.87	16.24
Sar + SE	30	90	26 (86.6%)	16 (61.5%)	7.95	9.21

### Saracatinib inhibits cognitive function deficits after SE

**Fig. 3:**
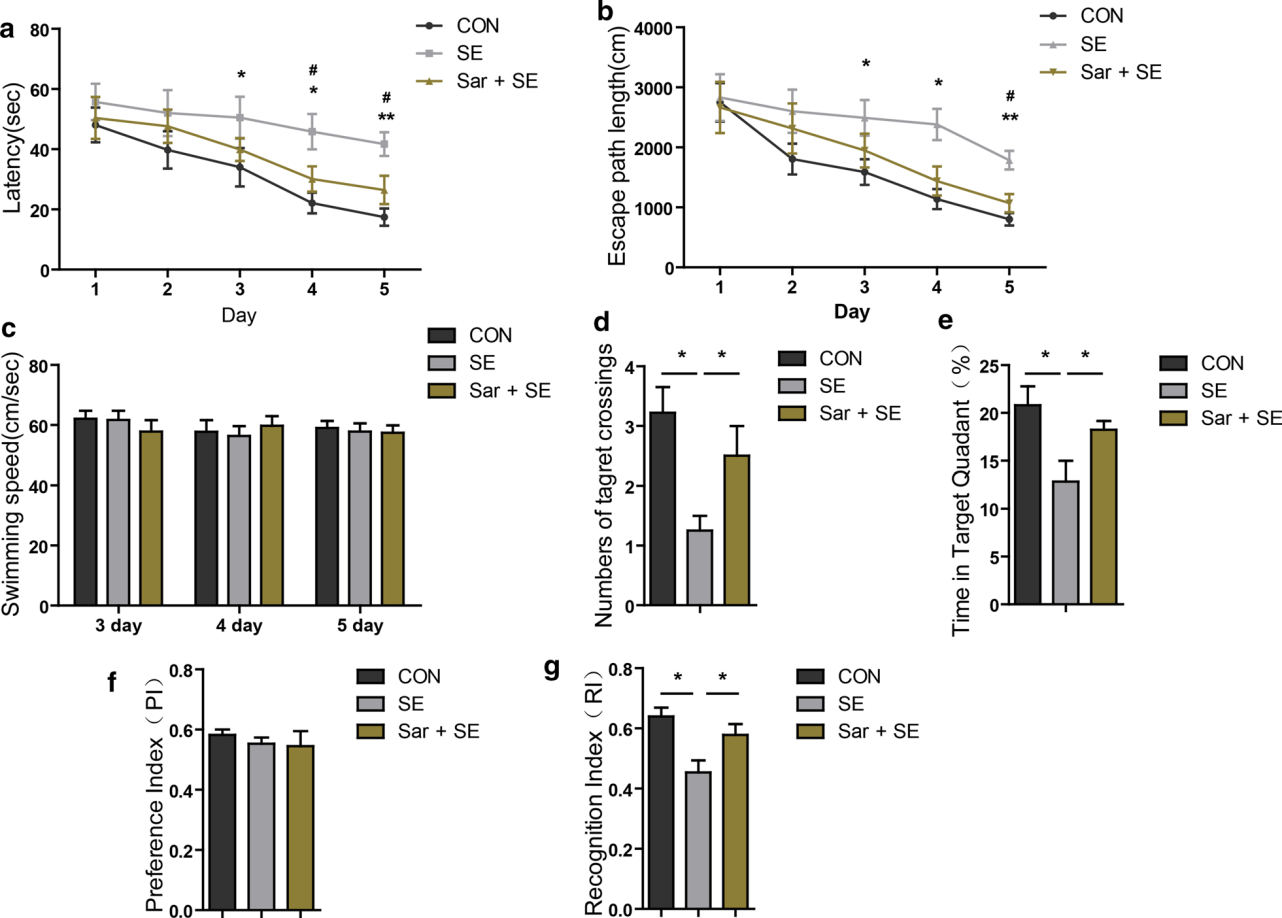
Saracatinib improves the cognitive function after status epilepticus. Cognitive function was analyzed using the Morris water maze. **a** Latency to the target platform during a five-day training period. **b** Distance swum to the target platform during a five-day training period. **c** Swimming velocity on days 3–5 during the training period. **d** The number of crossings of the platform site during the probe test. **e** Time spent in the quadrant of the platform placed in previous locations during the probe test. **f**, **g** The cognitive functions were analyzed using the normal object recognition test. **f** Preference index. (G) Recognition index. Data are expressed as the mean ± SEM (n = 8 or 9/group). *p < 0.05, **p < 0.01. * CON vs SE; # SE vs Sar + SE

Cognitive function deficits are a common sequelae after SE, so we examined whether saracatinib inhibited deficits in cognitive function after SE. Using the MWM test, escape latency (Fig. 3a) (F_(2,22)_ = 3.68; CON vs SE: 33.59 ± 3.8 vs 50.29 ± 4.87 on 3 days, *p* = 0.13; F_(2,22)_ = 7.116; CON vs SE vs Sar + SE: 22.13 ± 3.4 vs 45.83 ± 5.8 vs 30.11 ± 4.18 on 4 days, *p* = 0.01 in CON vs SE, *p* = 0.025 in SE vs Sar + SE; F_(2,22)_ = 10.307; CON vs SE vs Sar + SE: 17.42 ± 2.88 vs 41.72 ± 3.94 vs 26.45 ± 4.69 on 5 days, *p* = 0.009 in CON vs SE; *p* = 0.012 in SE vs Sar + SE) and swimming path length from the starting position to the target platform (Fig. 3b) (F_(2,22)_ = 3.02; CON vs SE: 1589.37 ± 213.03 vs 2490.87 ± 296.99 on 3 days, *p* = 0.23; F_(2,22)_ = 8.54; CON vs SE: 1138.7 ± 166.62 vs 2381.65 ± 258.46 on 4 days, *p* = 0.15; F_(2,22)_ = 13.81; CON vs SE vs Sar + SE: 802.907 ± 103.21 vs 1786.48 ± 155.98 vs 1070.8637 ± 113.52 on 5 days, *p* = 0.001 CON vs SE; *p* = 0.16 SE vs Sar + SE) were decreased in Sar + SE mice, when compared with the SE mice. However, the swimming speed was similar in all groups mice (Fig. 3c) (F_(2,22)_ = 0.54; CON vs SE vs Sar + SE: 62.09 ± 2.69 vs 61.72 ± 3.02 vs 57.85 ± 3.79 on 3 days, *p* > 0.05; F_(2,22)_ = 0.475, CON vs SE vs Sar + SE: 57.46 ± 2.87 vs 55.94 ± 3.3 vs 59.62 ± 2.6 on 4 days, *p* > 0.05; F_(2,22)_ = 0.11, CON vs SE vs Sar + SE: 59.01 ± 2.36 vs 57.85 ± 2.7 vs 57.43 ± 2.46 on 5 days, *p* > 0.05). There results suggested that decreased escape latency and swimming path length in the Sar + SE group mice were not due to an impaired capacity of swim. When the target platform was removed in the probe trails, the number of crossing target platform areas (Fig. 3d) (F_(2,22)_ = 5.917; CON vs SE vs Sar + SE: 3.22 ± 0.43 vs 1.25 ± 0.25 vs 2.5 ± 0.5, *p* = 0.012 in CON vs SE; *p* = 0.04 in SE vs Sar + SE) and staying time in the quadrant (Fig. 3e) (F_(2,22)_ = 5.202; CON vs SE vs Sar + SE: 20.8 ± 1.97 vs 12.84 ± 2.14 vs 18.237 ± 0.922, *p* = 0.01 in CON vs SE; *p* = 0.38 in SE vs Sar + SE) where the target platform was previously located were increased in the Sar + SE group, when compared with the SE group. Furthermore, when we performed the NOR test, the PI was similar in both SE group mice and Sar + SE group mice (Fig. 3f) (F_(2,25)_ = 0.326; CON vs SE vs Sar + SE: 0.58 ± 0.017 vs 0.553 ± 0.02 vs 0.545 ± 0.049, *p* > 0.05), but the RI of the Sar + SE mice was increased (Fig. 3g) (F_(2,25)_ = 6.944; CON vs SE vs Sar + SE: 0.64 ± 0.029 vs 0.453 ± 0.039 vs 0.578 ± 0.036, *p* = 0.015 in CON vs SE; *p* = 0.023 in SE vs Sar + SE). Together, these results showed that saracatinib inhibited deficits in cognitive function after SE.

### Saracatinib inhibits the loss of neurons in the hippocampus after SE

**Fig. 4:**
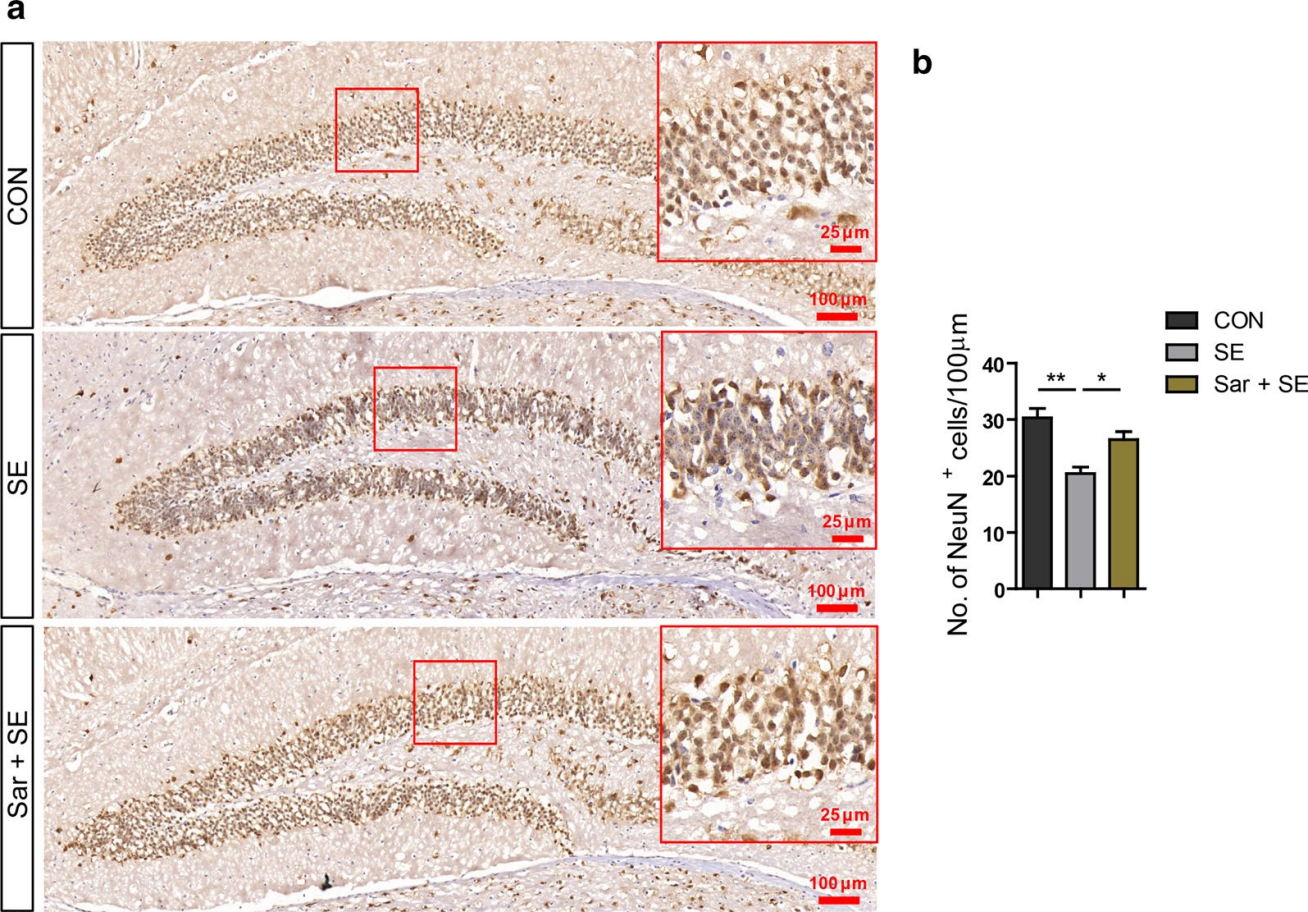
Saracatinib rescues the loss of hippocampal neurons after status epilepticus. **a** Immunohistochemical detection of NeuN^+^ neurons in the dental gyrus of the hippocampus. **b** Quantification of NeuN^+^ neurons in each group. Scale bar = 100 µm in lower magnification; = 25 µm in higher magnification insert. Data are expressed as the mean ± SEM (n = 5/group). *p < 0.05, **p < 0.01

Because hippocampal neuron loss is an important reason for the deficits in cognitive function after SE, we next determined the number of hippocampal neurons. Neurons in the DG region in the hippocampus were visualized by anti-NeuN antibody using immunohistochemical staining (Fig. 4a). The results showed that the number of neurons was increased in the DG in the hippocampus after saracatinib treatment than the SE groups (Fig. 4b) (F_(2,12)_ = 12.32; CON vs SE vs Sar + SE: 30.32 ± 1.65 vs 20.48 ± 1.09 vs 26.44 ± 1.42, *p* = 0.005 in CON vs SE; *p* = 0.032 in SE vs Sar + SE), suggesting that saracatinib relieved the cognitive function deficits by rescuing hippocampal neuronal loss during SE.

## Discussion

SE is a neurological condition with high mortality, involving continuous seizure activity, which is often considered as one of the precipitating factors for temporal lobe epilepsy (TLE). The hippocampus and piriform cortex are brain areas commonly damaged by SE. In addition, the amygdala, thalamus, neocortex, and cerebellum are also brain areas that are usually damaged after SE [21]. Neuronal loss in damaged brain areas, especially in the hippocampus, is the most common pathological change in TLE patients and animal models of SE [2, 22], which often contributes to cognitive function deficits [23, 24]. Rescuing the loss of hippocampal neurons is an important treatment to improve the cognitive function in patients with TLE. Pilocarpine-induced SE is a useful model for studying TLE because it has similar characteristics to human TLE. Here, we showed that saracatinib treatment effectively decrease the deficits in cognitive function in a pilocarpine-induced SE mouse model.

The Src kinase family is a family of non-receptor tyrosine kinases that plays important roles in regulating signal transduction. Fyn, as a member of the Src family kinases, plays critical roles in regulating cognitive function in AD and in frontotemporal dementia (FTD) patients [25, 26]. Fyn is widely expressed in the hippocampus [27] and is closely related to dendritic spine development and maintenance is critical for synaptic plasticity, responsible for cognitive function [7]. Fyn knockout mice exhibit decreased spine density in pyramidal neurons in the cerebral cortex [28], reduced axonal branching in granule cells in the cerebellar cortex [29], and spatial learning impairment [30]. Overexpression of Fyn also accelerated impairment of cognitive function in an AD mouse model [10, 31]. Cognitive deficits also occur in patients with seizures, except for patients with AD and FTD. However, the role of Fyn in regulating cognitive function after SE is not fully known. In the present study, we showed that the Fyn inhibitor, saracatinib, inhibited the deficit in cognitive function induced by SE using the MWM and NOR tests. MWM is the classic behavioral test to assess cognitive function. Spatial memory and learning were gained by finding the target platform during the training period. When the target platform was removed, mice with impaired spatial memory and learning had difficulty locating the area where the target platform was previously placed. Our results confirmed that saracatinib treatment improved spatial memory and learning. NOR is another behavioral test to assess cognitive function. It is the nature of mice to show interest in novel objects. Mice gained short-term memory for the identical object after the training phase, which helped to identify the novel object, so that more time could be spent with it. Saractinib-treated mice exhibited a higher RI in our study, which suggested that saracatinib improved short-term memory in mice after SE.

Loss of hippocampal neurons is the most common reason for cognitive deficits. Neuronal loss frequency occurs in both patients with TLE and in animal models of SE [2, 22, 32]. SE induces neuronal cell necrosis, apoptosis, and degeneration [33, 34]. Calcium (Ca) overload, autophagy, oxidative stress, and neuroinflammation produced during development and maintenance of SE are important inducing factors to stimulate these processes [35–38]. A previous report indicated that saracatinib reduced a-synuclein propagation to rescue the loss of dopaminergic neurons in the substantia nigra, to inhibit progressive Parkinson’s disease [39]. In the present study, we showed that saracatinib successfully rescued the loss of hippocampal neurons, although the mechanism responsible for this effect is unknown, which is worth investigating in further studies. Dendritic spines involving abnormal and synaptic plastic impairments in hippocampal neurons are also critical for cognitive function. Dysfunction of Fyn activity has been linked in both β-amyloid (Aβ) and tau pathology, which is responsible for the impairment of cognitive function in AD patients [40, 41]. Fyn may regulate some of the physiological and pathological functions, and it is a key mediator of Aβ toxicity. In the AD mouse model, Fyn accelerated synaptic and cognitive impairment, and rescued synaptic degeneration and memory loss when Fyn was depleted or its activity was inhibited [9, 10, 31]. In the present study, we therefore could not exclude the possibility that improvement in the cognitive function of post-SE mice also resulted from rescuing dendritic spines and synapses after sacaratinib treatment. This is worth investigating in further studies.

## Conclusions

The Fyn inhibitor, saracatinib, decreased cognitive deficits by attenuating the loss of hippocampal neurons in a pilocarpine-induced SE mouse model, indicating that inhibition of Fyn activity can potentially improve cognitive deficits in patients with TLE. However, further investigation is necessary to identify the mechanism responsible for this neuroprotective effects.

## Data Availability

The datasets used and/or analyzed during the current study available from the corresponding author on reasonable request.

## Abbreviations

SE: Status epilepticus
AD: Alzheimer’s disease
MWM: Morris water maze
NOR: Novel objection recognition
PBS: Phosphate-buffered saline
TLE: Temporal lobe epilepsy
FTD: Frontotemporal dementia
EEG: Electroencephalogram
DG: Dentate gyrus
PI: Preference index
RI: Recognition index
